# A model for predicting fall risks of hospitalized elderly in Taiwan-A machine learning approach based on both electronic health records and comprehensive geriatric assessment

**DOI:** 10.3389/fmed.2022.937216

**Published:** 2022-08-09

**Authors:** Wei-Min Chu, Endah Kristiani, Yu-Chieh Wang, Yen-Ru Lin, Shih-Yi Lin, Wei-Cheng Chan, Chao-Tung Yang, Yu-Tse Tsan

**Affiliations:** ^1^Department of Family Medicine, Taichung Veterans General Hospital, sTaichung, Taiwan; ^2^School of Medicine, National Yang Ming Chiao Tung University, Taipei, Taiwan; ^3^Department of Post-baccalaureate Medicine, College of Medicine, National Chung Hsing University, Taichung, Taiwan; ^4^School of Medicine, Chung Shan Medical University, Taichung, Taiwan; ^5^Institue of Health Policy and Management, National Taiwan University, Taipei, Taiwan; ^6^Department of Occupational Medicine, Taichung Veterans General Hospital, Taichung, Taiwan; ^7^Department of Computer Science, Tunghai University, Taichung, Taiwan; ^8^Department of Informatics, Krida Wacana Christian University, Jakarta, Indonesia; ^9^Center for Geriatrics and Gerontology, Taichung Veterans General Hospital, Taichung, Taiwan; ^10^Research Center for Smart Sustainable Circular Economy, Tunghai University, Taichung, Taiwan

**Keywords:** machine learning, elderly, prediction model, comprehensive geriatric assessment, fall accident

## Abstract

**Backgrounds:**

Falls are currently one of the important safety issues of elderly inpatients. Falls can lead to their injury, reduced mobility and comorbidity. In hospitals, it may cause medical disputes and staff guilty feelings and anxiety. We aimed to predict fall risks among hospitalized elderly patients using an approach of artificial intelligence.

**Materials and methods:**

Our working hypothesis was that if hospitalized elderly patients have multiple risk factors, their incidence of falls is higher. Artificial intelligence was then used to predict the incidence of falls of these patients. We enrolled those elderly patients aged >65 years old and were admitted to the geriatric ward during 2018 and 2019, at a single medical center in central Taiwan. We collected 21 physiological and clinical data of these patients from their electronic health records (EHR) with their comprehensive geriatric assessment (CGA). Data included demographic information, vital signs, visual ability, hearing ability, previous medication, and activity of daily living. We separated data from a total of 1,101 patients into 3 datasets: (a) training dataset, (b) testing dataset and (c) validation dataset. To predict incidence of falls, we applied 6 models: (a) Deep neural network (DNN), (b) machine learning algorithm extreme Gradient Boosting (XGBoost), (c) Light Gradient Boosting Machine (LightGBM), (d) Random Forest, (e) Stochastic Gradient Descent (SGD) and (f) logistic regression.

**Results:**

From modeling data of 1,101 elderly patients, we found that machine learning algorithm XGBoost, LightGBM, Random forest, SGD and logistic regression were successfully trained. Finally, machine learning algorithm XGBoost achieved 73.2% accuracy.

**Conclusion:**

This is the first machine-learning based study using both EHR and CGA to predict fall risks of elderly. Multiple risk factors of falls in hospitalized elderly patients can be put into a machine learning model to predict future falls for early planned actions. Future studies should be focused on the model fitting and accuracy of data analysis.

## Introduction

The world's population is aging rapidly (1). According to the United Nations, in 2019, 703 million people were aged 65 years or over worldwide with the number kept increasing (2). Taiwan is one the most rapidly aging countries in the world (3). In 2018, 14% of its people were in the aged population and this proportion will reach 20% by 2025, or equivalent to taking only 7 years to switch from an aged society to a super-aged society. Such rapid change causes large amounts of burdens, whether physical, psychological or social (4–6).

Fall is one of the most important concerns in the elderly population, and it is a key geriatric syndrome (7, 8). Over 1/4 of these old people experience falls every year (9). Falls have multiple devastating consequences, both physically and psychologically. Examples include hip fracture (10), head trauma (11), depression, social isolation and loneliness (12), disability (13) and even death (14).

Many important risk factors have been identified among those elderly experiencing falls. Internal risk factors include multimorbidity (15), sarcopenia (16), frailty (17), polypharmacy (18), inappropriate medication (19), malnutrition (20), poor visual acuity (21) and hearing impairment (22). External risk factors include inappropriate clothes, inappropriate shoes, inadequate light, obstacles on the ground (23). Caregivers are also important external risk factors. Kuzuya et al. (24) found that falls were associated with caregiver burden even when controlling for various possible confounding factors. Mamani et al. (25) discovered that caregivers knew about falls and its prevention, but in a superficial way, and it's important to influence their attitudes and practices regarding the prevention of fall.

The prediction of fall risks is essential especially for those healthcare professionals caring for the elderly. Many prediction methods have been proposed, such as Morse Fall Scale (26), STRATIFY Scale (27) and Hendrich Scale (28). Another tool is the short physical performance battery (SPPB) which assesses fall risk by measuring balance, gait, and muscular strength (29). However, these scales do not capture all possible fall risk factors and increase work loads of healthcare professionals in data collection and analysis.

With advancing technology and improved medical informatics, some researchers predicted falls in hospitalized patients based on electronic health records (EHR), but data from HER also have some limitations (30, 31). Since many risk factors have been found and the evolution of computer science and artificial intelligence, many scientists would like to predict falls by means of machine learning (32–35) (Table 1). However, most datasets consisted of relatively healthy people, or young people (36). Furthermore, few of such research have analyzed Asian populations, which have very different socio-economic profiles compared with the western populations. Here, we aimed to build up a fall risk prediction model for the hospitalized elderly based on machine learning, using a combination of EHR and comprehensive geriatric assessment (CGA).

**Table 1:** Previous researches regarding fall risk prediction by machine learning.

**Reference**	**Number of predicted variables**	**Machine learning model**	**Outcome**
Lindberg et al. (34)	38	Classification tree, bagging, random forest, and adaptive boosting methods	In terms of AUROC, bagging (0.89), random forest (0.90), and boosting (0.89) all outperformed the Morse Fall Scale (0.86) and the classification tree (0.85)
Oshiro et al. (32)	13	Logistic regression	Sensitivity of 67%, specificity of 69%, positive predictive value of 8%, negative predictive value of 98%, and area under the curve of 0.74
Jung et al. (33)	165	Logistic regression, Cox PH regression, and decision tree algorithms	In terms of AUROC, logistic regression (0.86), Cox PH regression (0.75), and decision tree (0.73)
Liu et al. (35)	54	Decision tree, Bayesian network, support vector machine, and random forest	Bagging + RF classifier generated the optimal prediction results for all four points during the inpatient hospitalizations: within 24 h of hospital admission (1d, AUC = 0.71), after 24 h of hospital admission (1st, AUC = 0.72), the maximum value dataset within multiple assessment patient records (max, AUC = 0.74), and the last recorded patient datasets (last, AUC = 0.76).

## Materials and methods

### Dataset

Our research dataset was provided by the Clinical Data Center of Taichung Veterans General Hospital. We enrolled all elderly who were admitted to our geriatric care unit during the period from January 1, 2018 to December 31, 2019. During hospitalization, we collected patients' data regarding their general demographic data, medical history, blood examination, medication information, and CGA. Multiple assessments were performed in CGA for the elderly, including physical evaluation, psychological evaluation, and social evaluation. The parameters of CGA included the patients' demographic information, including age, gender, body mass index (kg/m2), education level, marital status, decision-making individual, caregiving support, and measurement data. The measurement data involved cognitive impairment (defined as scores <24 on the Chinese version of the Mini-Mental State Examination, MMSE), mood disorder (defined by scores ≥2 on the 5-item Chinese Geriatric Depression Scale, GDS-5), medical condition (defined by the Charlson comorbidity index, CCI), polypharmacy (defined as currently using >4 drugs), psychiatric medication (defined as using any antipsychotics, antidepressants or benzodiazepines during admission), malnutrition (defined by scores <12 on the Mini-Nutritional Assessment-Short Form, MNA-SF), physical function (assessed by the Barthel index of Activities of Daily Living, ADL and the Lawton Instrumental Activities of Daily Living Scale, IADL), health-related quality of life (measured by the Chinese version of the EQ-5D system), as well as frailty in accordance with Fried's definition of the frailty phenotype, which was evaluated based upon the presence of three or more criteria: weight loss, low physical activity, exhaustion, weakness (hand grip strength), and slowness (walking speed). In order to avoid redundant data collection from the same person, for those with multiple hospitalization data, only data from the latest hospitalization were retrieved. The final dataset contained a total of 1,115 patients with non-redundant data. Regarding fall incidence, the record of falls was derived from the CGA questionnaire. After the two datasets are merged according to the de-identified ID, we obtained a total of 1,101 records. The study was conducted according to the guidelines of the Declaration of Helsinki, and approved by the Institutional Review Board (or Ethics Committee) of Taichung Veterans General Hospital (protocol code TCVGH-IRB CE20234A and date of approval: Aug 13, 2020).

### Machine learning and prediction model development

We used 6 different models to predict fall among elderly. These models included algorithms of random forest, XGBoost, Logistic regression, LightGBM, SDG and DNN.

#### Random forest

Random Forest belongs to Ensemble Learning. It is an advanced version of decision tree. It consists of multiple decision trees, but there is no relationship between different decision trees. During classification, each new sample will be judged and classified by each decision tree in the forest, and each decision tree will get a classification result. Finally, the random forest gathers all the classification voting results, and counts the number of votes. The highest category is designated as the final result.

The RF equation is as follows:


$$\begin{array}{l} RF=\overset{K}{\underset{k=1}{\sum }}p_{k}\left(1-p_{k}\right)=1-\overset{K}{\underset{k=1}{\sum }}{p_{k}}^{2} \end{array}$$

#### XGBoost

The full name of XGboost is Extreme Gradient Boosting (Extreme Gradient Boosting). It keeps the original model unchanged in each operation, and then adds a new function to the model, so that the tree generated later can correct the errors of the previous tree. In addition, XGBoost uses random feature extraction when generating trees, so all features will not be used in decision-making every time in tree generation.

The equation of XGBoost is as follows:


$$\begin{array}{l} XGBoost=\overset{n}{\underset{i=1}{\sum }}l\left(y_{i},y_{i}^{\left(t\right)}\right)+\overset{t}{\underset{i=1}{\sum }}\Omega \left(f_{i}\right) \end{array}$$

#### Logistic regression

The logistic regression model is a type of linear classifier, which is mainly used in binary classification problems. It is mainly to classify according to the data it has, and to judge the data to determine what category the data belongs to. The output value of the logistic regression model classification needs to be in [0,1].

#### LightGBM

LightGBM is a gradient boosting framework that uses tree-based learning algorithms. Algorithms supported by the LightGBM framework include: GBT, Gradient boosting decision tree (GBDT), Gradient Boosted Regression Trees (GBRT), Gradient Boosting Machine (GBM), Multiple Additive Regression Trees (MART), and Random Forest (RF). Sparse optimization, parallel training, various loss functions, regularization, bagging, and early halting are some features that LightGBM has over XGBoost. The structure of trees is a significant difference between the two. Unlike most other implementations, LightGBM does not grow a tree row by row. Rather, it grows trees leaf-by-leaf. It selects the leaf that it considers giving the greatest reduction in loss. Furthermore, unlike XGBoost and other implementations, LightGBM does not adopt the commonly used sorted-based decision tree learning algorithm, which searches for the optimum split point of sorted feature values. Furthermore, LightGBM offers a proprietary optimized histogram-based decision tree learning algorithm, which provides good performance and memory savings. Gradient-Based One-Side Sampling (GOSS) and Exclusive Feature Bundling (EFB), two unique techniques used in the LightGBM algorithm, provide modeling with higher speed of execution together with better performance accuracy (37).

#### DNN

A neural network is a mathematical computing model that imitates the construction of biological neural networks in the field of machine learning. Neural networks perform computations by connecting a large number of neurons to each other.

### Data pre-processing

Given the constraint of too many relevant risk factors in establishing an accurate model, it is necessary to screen out the more relevant factors first through data pre-processing. For data classification, insufficient accuracy is likely a problem. In this part, the accuracy needs to be improved through testing and changes of different algorithms. In the model selection of deep learning and machine learning, the feedback of various models to different data factors is considered. Through multi-party testing, while avoiding the problem of overfitting or underfitting of the model to the data, a good model needs to adjust itself or initiate “Early Stopping” and other procedures for proper adjustments.

Spearman's correlation, Pearson's correlation and Kendall's correlation coefficient were used to analyze all features included in the study to show the correlations of each factors.

Since the physiological values have different range intervals in the data set, it is necessary to normalize them and map their values to (0,1) intervals. This normalization improves the convergence speed during model training. The rest of the past medication data and symptom descriptions are encoded by One-Hot Encoding, using 1 and 0 to replace the parameters that are not represented in numbers.

### Data analysis by machine learning

Physical examination variables of 1,101 elderly patients were subsequently analyzed. A total of 21 potential factors were used to predict the probability of accidental misses of the elderly in the future. An expert group consisting of geriatrician, clinical physician, professor in informatics and data analyst was gathered before the study. We had regular meeting with members of the expert group, each feature was viewed and discussed by all members and selected from previous experience and research. Data of 1,101 elderly subjects were divided into three sets: training, validation, and test, at a ratio of 3:1:1. The reason why we chose three sets was because we could perform validation and testing after training immediately, and this could increase the effectiveness and usefulness of the model. Previous study also used three sets in detection of glaucomatous optic neuropathy (38). Another study also used three sets design to distinguish endometrial cancer among 926 patients (39).

Several methods are used to evaluate machine learning models. Here, we divide the data set to be used for machine learning into the training data and testing data. For validation data, the training data set is used to train the model, and the validation data set is used to evaluate the training model. Therefore, we can pay attention to the status of model training in real time. Once the model is trained and verified, it can be used for inferencing the model. The final test is performed on the test data set, so that the performance accuracy of the machine learning model can be better revealed.

In this research, we used two approaches to generate the data set. The first approach is a simple cutting of a data of set, i.e., a data set is cut from two face-image data sets, and divided at a ratio of 3:1:1 into the training set, test set, and validation set. At the end of training, the test set that has not been used in the training is fed as input to the model for prediction. The result serves as model one reference for evaluating the accuracy of model prediction.

The second approach is cross validation. During training of machine learning, the training set, validation set and test set are cut at a ratio of 3:1:1 or 7:3. Cross-validation is a method from statistics. In order to avoid errors caused by the model's excessive dependence on specific training and validation sets, the parent data set is cut into a greater number of subsets to allow different combinations of data sets. Some subsets are first arbitrarily selected as training set and validation set. In the next training, different subsets are selected as training set and validation set to minimize modeling errors.

The K-fold cross validation is the most classic and most commonly used method. The K of K-fold is the same as the K of K-mean and KNN, which refers to one Number, a number that can be defined by the user. Figure 3 shows the K-fold verification flow chart when K is equal to 5. We divide the data into three equal parts, the first part is used as the test data for verification, and the remaining two with one copy used for training. In the next round, the second aliquot is used again as the test data for verification, and use the other two for training. After three such rounds, the accuracy results of the three modeling exercises are averaged, or to return to the evaluation index of the problem. The average value provides a fair estimate of the model performance on the overall data set.

Activation function is a non-linear function, to allow neural network models to deal with non-linear features. Commonly used functions are ReLu, Sigmoid (binary classification), Tanh (binary classification), the Softmax (multivariate classification) or the function for ReLu, which we used in this study. It is also known as a linear function of rectifiers like in Supplementary Figure 1. In fact, it is a maximum value function. When the input is <0, the output is 0. When the input is >0, the output is equal to the input. The advantage of the ReLu function is its fast convergence speed compared with the Sigmoid and Tanh functions. When the input is positive, it overcomes the problem of disappearing gradient, but it has disadvantages similar to Sigmoid function. The output of ReLu is not zero-centered. When the input is negative, ReLu is complete. If it is not enabled, it means that as long as the input is a negative number, ReLu will not function.

The full name of the RMSProp optimization algorithm is Root-Mean-Square Prop. The adaptive algorithm proposed by Geoff Hinton updates and changes the iterative calculation according to the gradient and error of its calculated parameters. The calculation formula of RMSProp is as follows:


$$\begin{array}{l} {{\;x}_{i}}^{t+1}={x_{i}}^{\left(t\right)}-\frac{\eta }{\sqrt{E{\left[g^{2}\right]}_{t}}}\frac{\delta C}{\delta w}\\ E{\left[g_{i}^{2}\right]}_{t}=\rho E{\left[g_{i}^{2}\right]}_{t-1}+\left(1-\rho \right)g_{t,i}^{2} \end{array}$$

*x*_*i*_^(t)^: Indicates the parameter updated for the tth time.

γ: Represents the learning rate.

*g*_*t, i*_: indicates the gradient of the first number.

ρ*t***–**: Represents the weight of the gradient average of the past t1 time, usually set to 0.9.

E[]: The expected value of the value.

ε: The deviation value to be corrected.

Accordingly, RMSProp introduces a coefficient, which decreases each time at a certain ratio, so that the learning rate can be scaled and corrected based on the formula. Compared with the cumulative square gradient Adagrad, RMSprop calculates the corresponding average value, and alleviates the problem of the Adagrad in its fast drop in learning rate. Hence, the momentum adjustment of RMSProp is better than that of AdaGrad. For convolutional neural networks, an algorithm that can adjust momentum parameters is undoubtedly a good choice. RMSProp is known to be a practical and effective deep learning network optimizer in practical applications and comparison tests.

Adam's name comes from Adaptive Moment Estimation, which combines Adagrad and RMSProp and performs correction of deviation terms. It retains the learning rate of RMSProp to calculate the adaptive parameters based on the average value of the first-order matrix, and also makes full use of the average value of the second-order matrix of the gradient, thereby controlling the attenuation rate. Adam's calculation formula is as follows:


$$\begin{array}{l} x^{\left(t+1\right)}=x^{\left(t\right)}-\frac{\gamma }{\sqrt{{\hat{\nu }}_{t}+\varepsilon }}{\hat{m}}_{t}\\ \;{\hat{m}}_{t}=\frac{m_{t}}{1-\beta_{1}{}^{t}}\\ \;{\hat{\nu }}_{t}=\frac{\nu_{t}}{1-\beta_{2}{}^{t}} \end{array}$$

*x*^(*t*)^: Indicates the i-th updated parameter.

γ: Represents the learning rate.

*m*_*t*_: Represents the first-order momentum difference function of the gradient.

*v*_*t*_: Represents the gradient second-order momentum difference function.

β_1_: Adjustable parameter, usually set to 0.9.

β_2_: Adjustable parameters, usually set to 0.999.

ε: The deviation value to be corrected, usually set to 10^−8^.

When the gradient matrix is sparse, the application of Adam's second-order momentum difference and the correction of its deviation value allows it to perform faster than the RMSProp algorithm. Therefore, in the absence of special circumstances or requirements, Adam's method is typically the first choice.

After settings of the startup function, loss function and optimizer, one can adjust the number of neural layers of the machine learning model and the number of neurons at each neural network layer. Generally speaking, adding more neural layers and neurons for model training based on the number of elements, the more features can be learned. But it is also more likely to over fitting. At this time, the regular processing needs to be used, as will be described later, like the discarding method, and L1 and L2 conventional methods.

The mathematics behind the normalization is to add a normalized term after the original loss function, the purpose is to generate a smoother function.


$$\begin{array}{l} L1:||w|{|}_{1}=\overset{m}{\underset{j=1}{\sum }}|w_{j}| \end{array}$$

L1 normalization is to take the absolute value of all the parameters in the model. Mathematically, because the absolute value cannot be differentiated, the difference of >0 is roughly differentiated as the derivative of 1, <0 is−1, as expressed by the sgn function.

After adding to the new loss function a term of normalization for the partial differentiation of each parameter wi, every time when updating the parameter wi, an ηλsgn(wi) will be deducted from the expression. Let the parameter wi be close to 0.

The model in the Supplementary Figure 2 is a linear regression. The blue is the contour line encountered during the optimization process. A circle represents an objective function value. The center of the circle is the sample concern value, the radius is the error value, and the restricted condition is the red boundary. The intersection of the two is the optimal parameter. The Figure shows that optimal parameters can only be on the coordinate axis, so there will be 0 weight parameters, making the model sparse.

L1 normalization simplifies the complexity of the model, sets those useless weights to 0, saving those weights that the model considers important. The sparse nature derived from L1 normalization has been widely used in feature selection mechanisms. Feature selection picks meaningful features from the available feature subset, simplifying machine learning problems.


$$\begin{array}{l} L2:||w|{|}_{2}^{2}=\sqrt{\overset{n}{\underset{i=1}{\sum }}X_{i}^{2}} \end{array}$$

L2 normalization is the sum of all the parameters in the model. Mathematically, the new loss function with the term of normalization is added. After the partial differentiation of the two parameters wi, each time with updating the parameter wi, it will be multiplied by (1–in front of wiηλ). Because the η heels λ are very small values, (1–ηλ) is about 0.99, which though <1, is very close to 1.

The model in the Supplementary Figure 3 is a linear regression. The blue is the contour line encountered during the optimization process. A circle represents an objective function value. The center of the circle is the sample concern value, the radius is the error value, and the restricted condition is the red boundary. The intersection of the two is the optimal parameter. From the Figure, it can be seen that the optimal parameters can only be on the coordinate axis, achieving a balance between w1 and w2 and reducing over-fitting.

L2 normalization also simplifies the model, but instead of leaving only a certain weight, it weakens all weights and makes all weights and neurons active. Make the weight smaller each time, which is called weight decay, the L2 regularization mainly prevents model overfitting.

The hyperparameters used for best modeling were as follows: max_depth = 6, learning_rate = 0.300000012, subsample = 1, colsample_bylevel = 1, colsample_bytree = 1, min_child_weight = 1, gamma = 0, scale_pos_weight = 1.

## Results

Table 2 shows the demographic and clinical characteristics of 1,101 elderly patients, including 349 fallers and 752 non-fallers. Their mean age was 86.08 years old, with females predominant (60.4%). There were no significantly different among features between faller and non-fallers. However, fallers had lower ADL, lower IADL and more sleep disturbance than non-fallers, with significant difference.

**Table 2:** Demographics and clinical factors of participants.

	**Total (*n* = 1,101)**	**Fallers (*n* = 349)**	**Non-Fallers (*n* = 752)**	
**Patient characteristics**	***n* (%) / Mean**	***n* (%) / Mean**	***n* (%) / Mean**	***p*-value**
Male	436 (39.6%)	152 (43.6%)	284 (37.8%)	0.0677
Age	86.08	86.32	85.97	0.5339
Weight	57.83	57.09	58.17	0.1575
Height	157.65	157.03	157.94	0.1673
Diastolic pressure	67.9	68.08	67.82	0.7343
Systolic pressure	131.49	131.24	131.61	0.7972
Heart rate	80.04	79.95	80.08	0.8924
Respiratory rate	18.13	18.22	18.09	0.6421
ADL	42.03	38.67	43.6	0.0159^*^
MNA	18.45	18.12	18.6	0.1793
Brade score	18.08	17.84	18.19	0.0872
IADL	1.81	1.51	1.95	0.0012^*^
VAS	0.83	0.94	0.78	0.1907
CHS	2.54	2.63	2.5	0.2067
Polypharmacy	832 (75.6%)	276 (79.1%)	556 (74.0%)	0.0644
Psychiatric medication	400 (36.3%)	132 (37.8%)	268 (35.6%)	0.4832
Visual impairment	667 (60.6%)	209 (59.9%)	458 (60.9%)	0.7475
Hearing impairment	504 (45.8%)	164 (47.0%)	340 (45.2%)	0.5815
Difficulty in communication	367 (33.3%)	120 (34.4%)	247 (32.9%)	0.6144
Sleep disturbance	471 (42.8%)	165 (47.3%)	306 (40.7%)	0.0398^*^
Urinary incontinence	606 (55.0%)	206 (59.0%)	400 (53.2%)	0.0702

*ADL, Activity of daily living; IADL, Instrumental activity of daily living; MNA, Mini-nutritional assessment; VAS, Visual analog scale; CHS, Cardiovascular health study.*

* *P < 0.05*.

Table 3 shows the difference of accuracy, AUC, sensitivity and specificity among all models, including XGBoost, LightGBM, Random Forest, Logistic regression, SGD and DNN. In the deep learning algorithm, 5-layer neural networks are used for stacking (Supplementary Figure 4). After the experiment is completed, if the number of layers continues to be superimposed, it will cause the model to be overfitting. The fitting situation occurs, so this project only constructs a 5-layer neural network model for prediction, and the test set is used to verify the accuracy of 73.2%. When training the model for the second time, we removed the 10 least important factors in F-Score and retrained the model. But the model effect was almost the same as the first time, and the verification accuracy on the test set was again 73.2%.

**Table 3:** Accuracy, sensitivity, specificity, AUC and other predictive value of all prediction models.

	**Accuracy**	**Sensitivity**	**Specificity**	**AUC micro**	**f1 score micro**	**Precision score micro**	**Recall score micro**
XGBoost	73.2%	91.0%	26.0%	57.0%	73.2%	72.1%	72.1%
LightGBM	70.7%	69.4%	69.4%	69.4%	70.7%	69.4%	69.4%
RandomForest	73.0%	69.4%	69.4%	69.4%	73.0%	69.4%	69.4%
Logistic	70.2%	68.5%	58.5%	68.5%	70.2%	68.5%	58.5%
SGD	55.9%	53.2%	53.2%	53.2%	55.9%	53.2%	53.2%
DNN	65.6%	13.6%	35.9%	35.9%	19.7%	35.9%	35.9%

**Figure 1:**
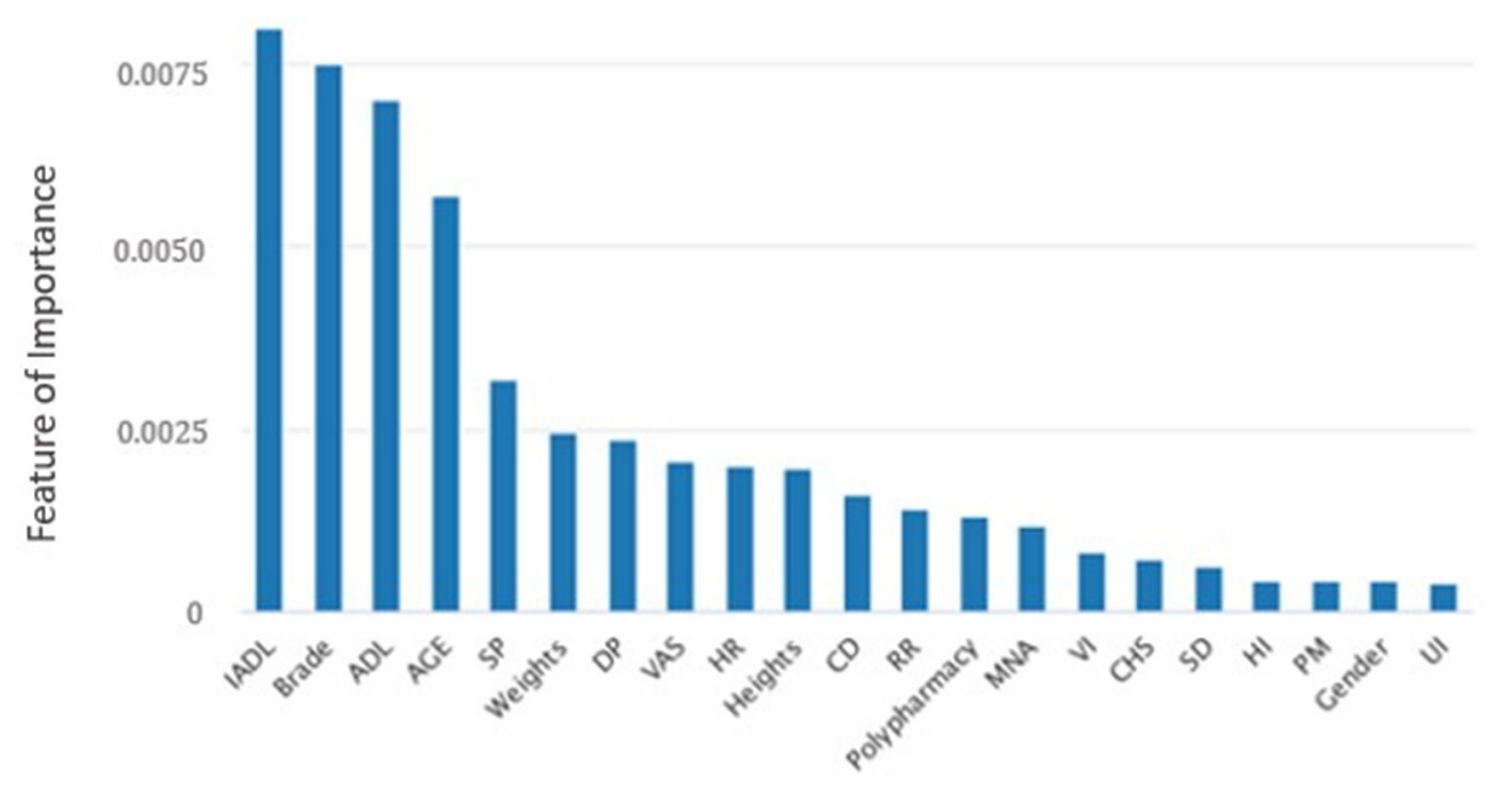
Feature of importance of all 21 features in XGBoost model. IADL, Instrumental activity of daily living; ADL, Activity of daily living; SP, Systolic pressure; DP, Diastolic pressure; VAS, Visual analog scale; HR, Heart rate; CD, Communication disturbance; RR, Respiratory rate; MNA, Mini nutritional assessment; VI, Visual impairment; CHS, Cardiovascular health study; SD, Sleep disturbance; HI, Hearing impairment; PM, Psychiatric medication; UI, Urinary incontinence.

**Figure 2:**
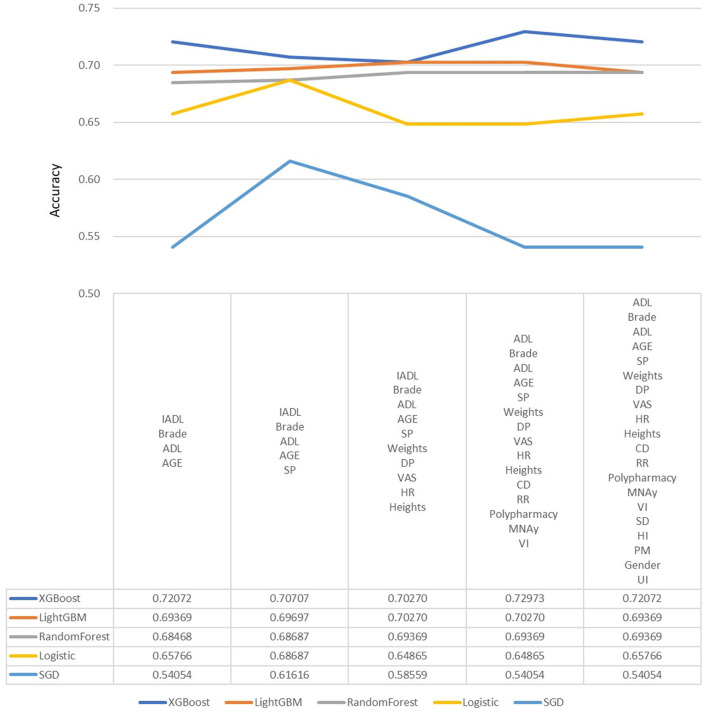
Predictive accuracy for combined different features in different machine learning models. DP, Diastolic pressure; ADL, Activity of daily living; HR, Heart rate; SP, Systolic pressure; IADL, Instrumental activity of daily living; CHS, Cardiovascular health study; MNA, Mini nutritional assessment; VAS, Visual analog scale; RR, Respiratory rate; CD, Communication disturbance; VI, Visual impairment; SD, Sleep disturbance; HI, Hearing impairment; UI, Urinary incontinence; PM, Psychiatric medication.

In the machine learning algorithm XGBoost, the maximum depth of each tree is set to 6 layers. Although increasing this value will complicate the model, it more likely over-fits. Classifying through the algorithm, the importance of the features in the classification process is calculated. From Figure 1, we found that IADL, Brade score, ADL, age and systolic pressure have a higher F-score compared to other feature factors. Results showed that these 5 features are the priority classification factors of the decision tree. Accuracy of multiple features in different prediction models are shown in Figure 2. It revealed that with only 15 features, XGBoost model gave the highest accuracy (73.0%). For the model XGBoost, we calculated 95% CI for accuracy, sensitivity, specificity and auROC (Supplementary Table 1). Independent testing was performed for XGBoost model (Supplementary Table 2). We've also calculated the predictive performance based on the top 5 features in XGBoost model, and the accuracy was 70.7% (Supplementary Table 3).

**Figure 3:**
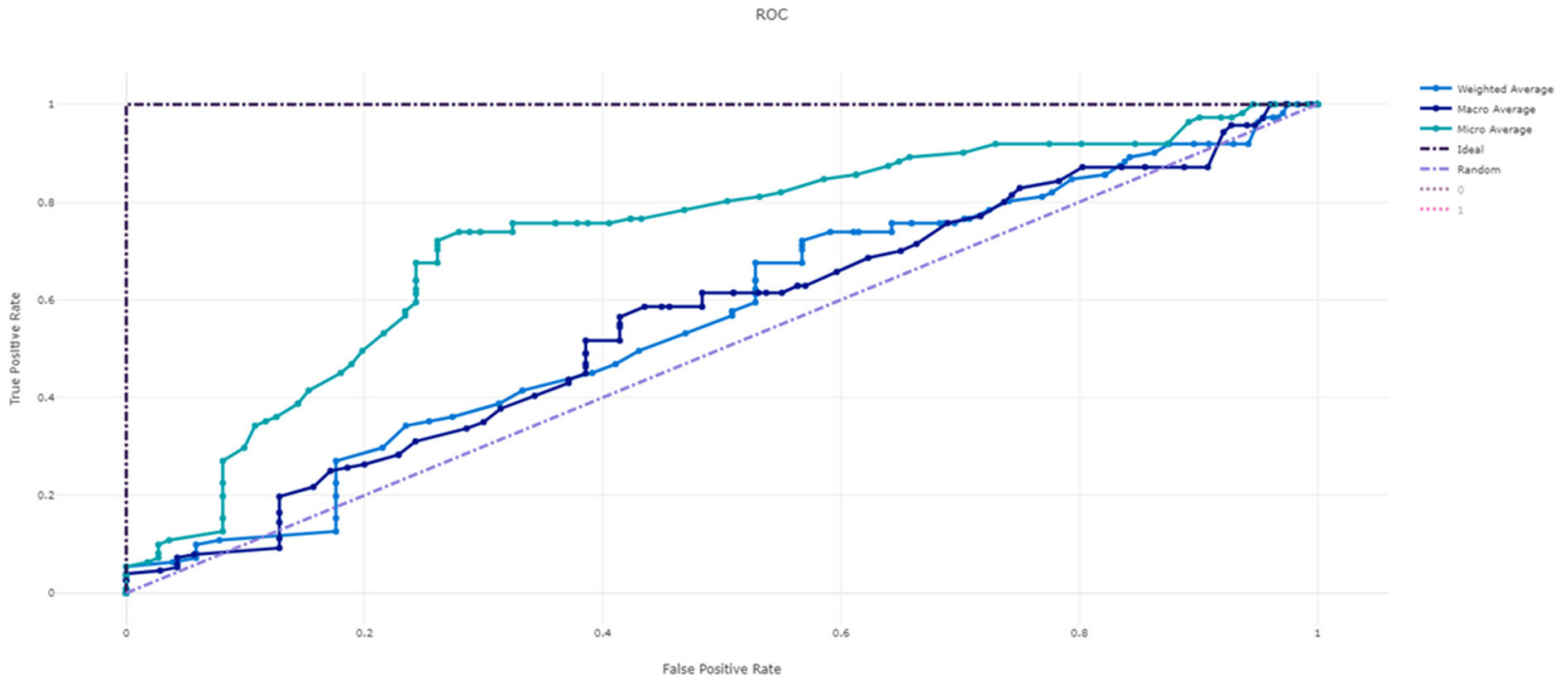
ROC curve of XGBoost model predicting fall risk among hospitalized elderly.

**Figure 4:**
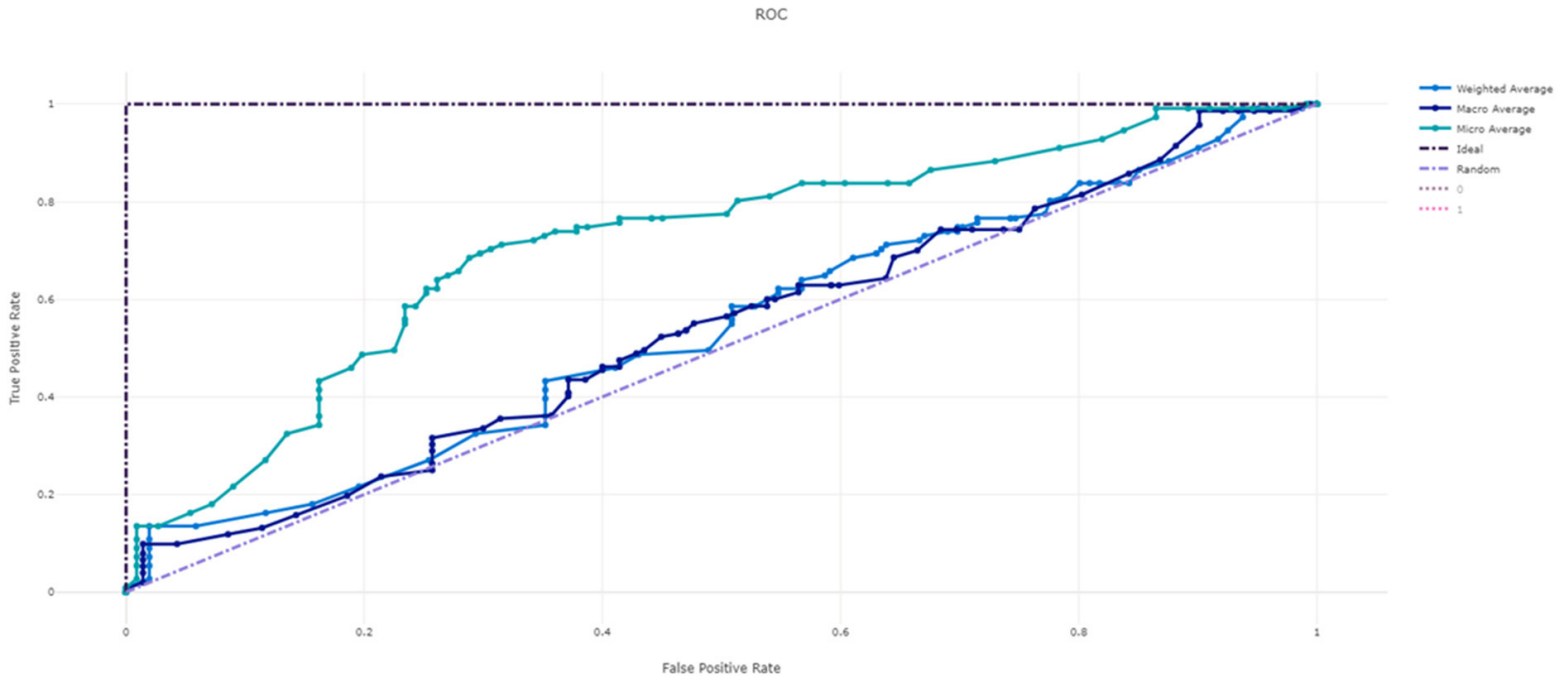
ROC curve of LightGBM model predicting fall risk among hospitalized elderly.

**Figure 5:**
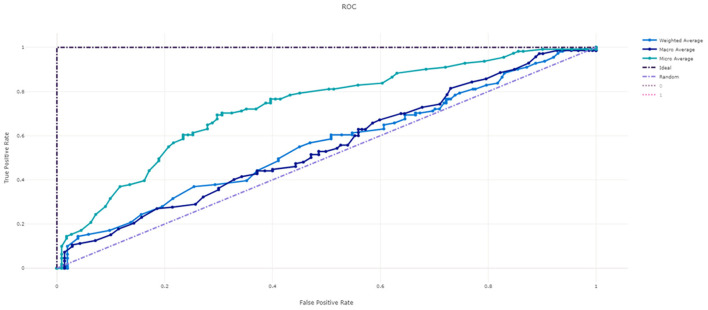
ROC curve of Random Forest model predicting fall risk among hospitalized elderly.

**Figure 6:**
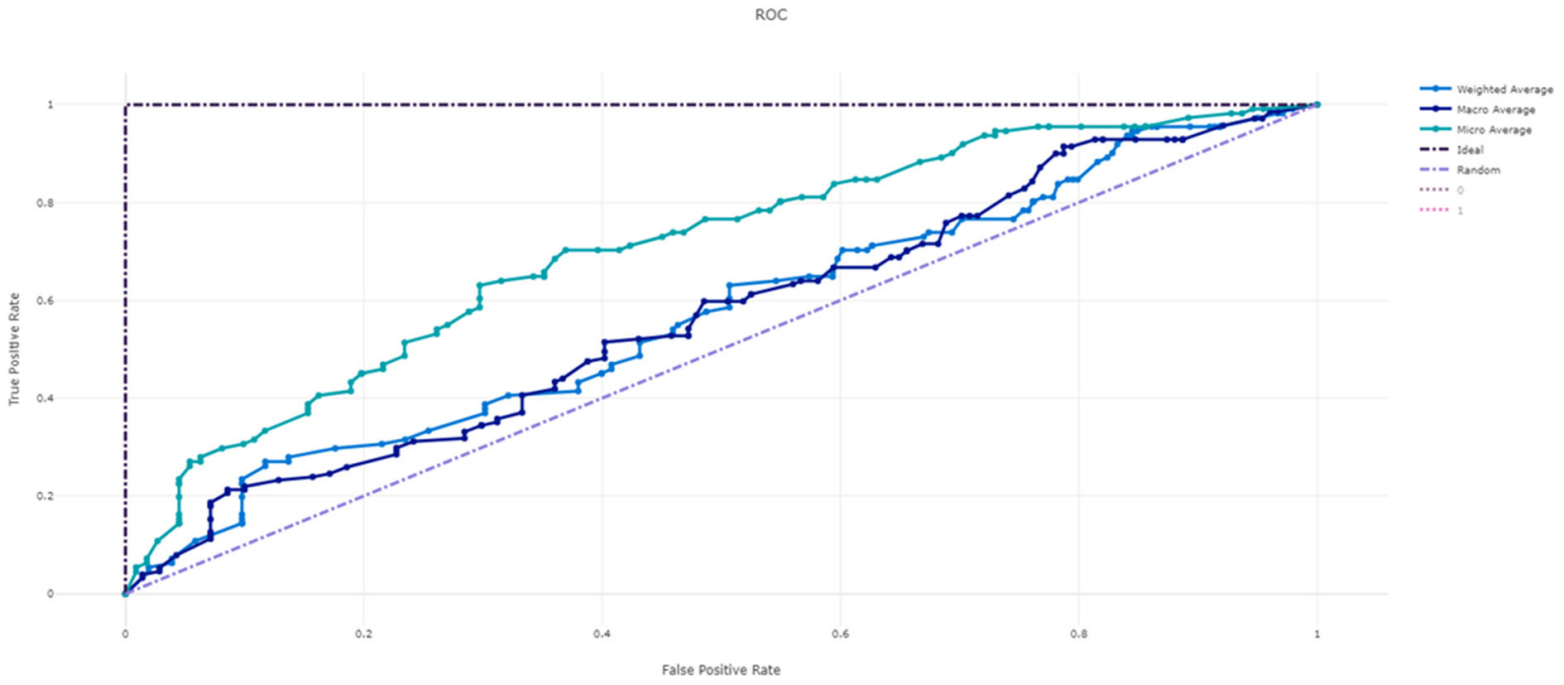
ROC curve of Logistic Regression model predicting fall risk among hospitalized elderly.

**Figure 7:**
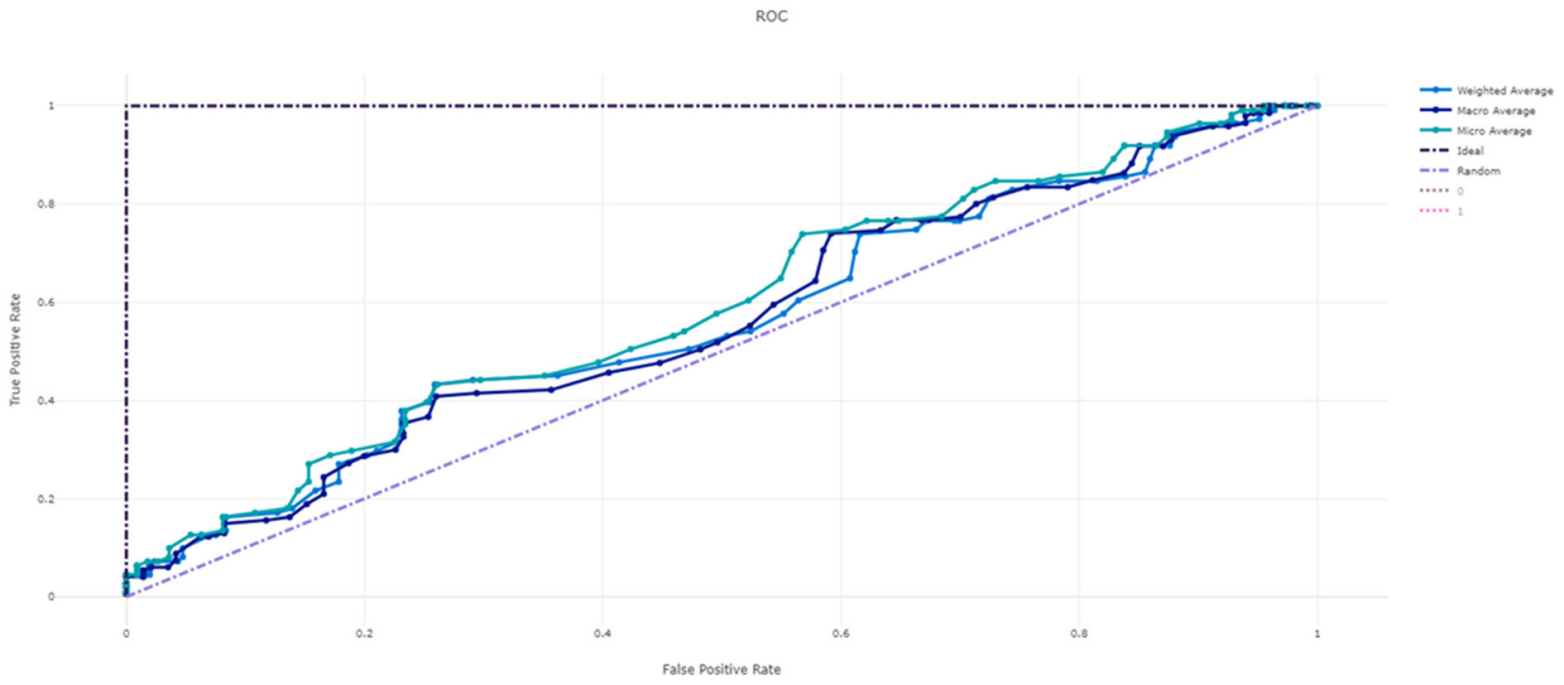
ROC curve of SGD model predicting fall risk among hospitalized elderly.

Figure 3 shows the ROC curve of XGBoost, and Figures 4–7 show ROC curve of LightGBM, RF, Logistic regression and SGD, respectively. Compared with other models, XGBoost had best auROC.

**Figure 8:**
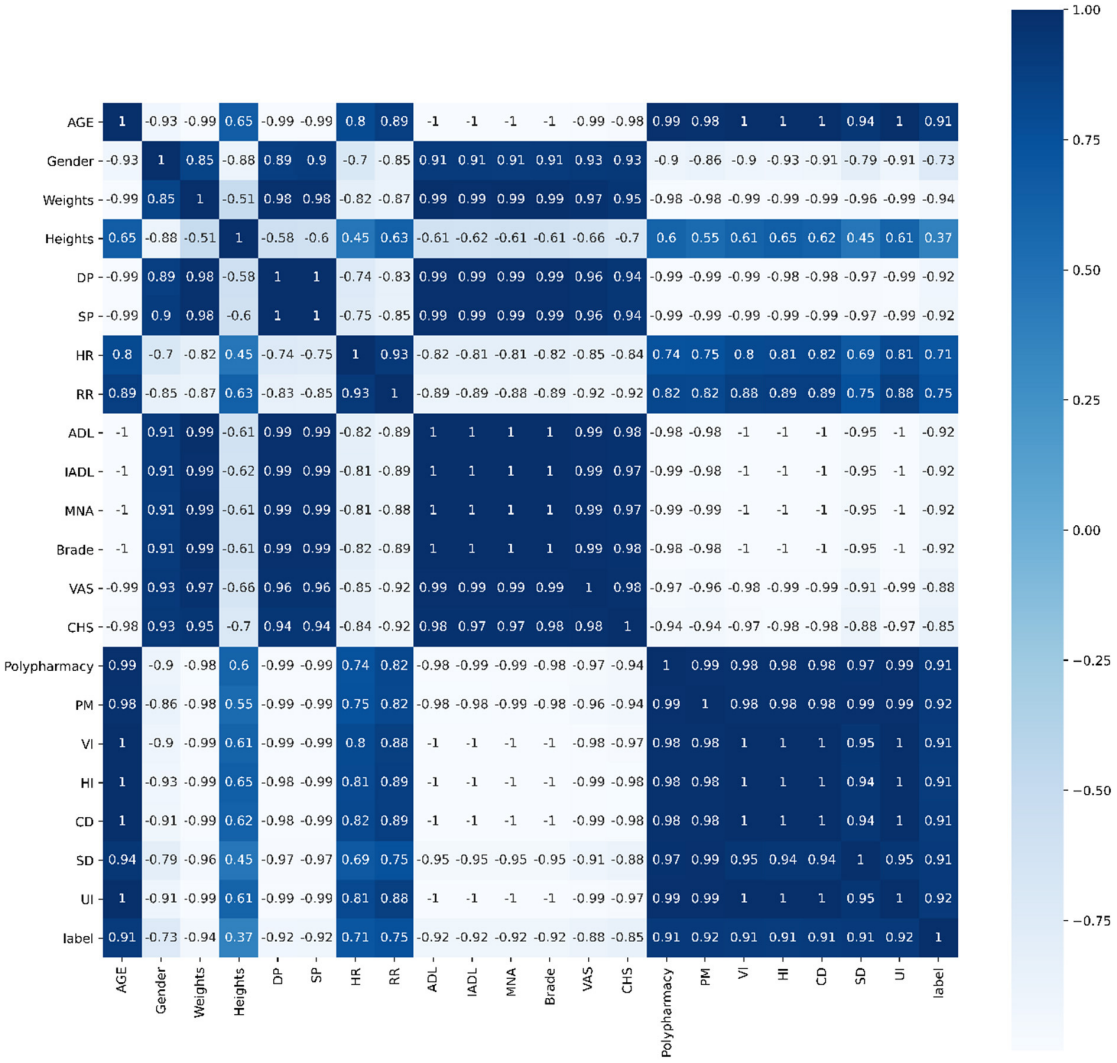
Pearson's correlation of all 21 features.

**Figure 9:**
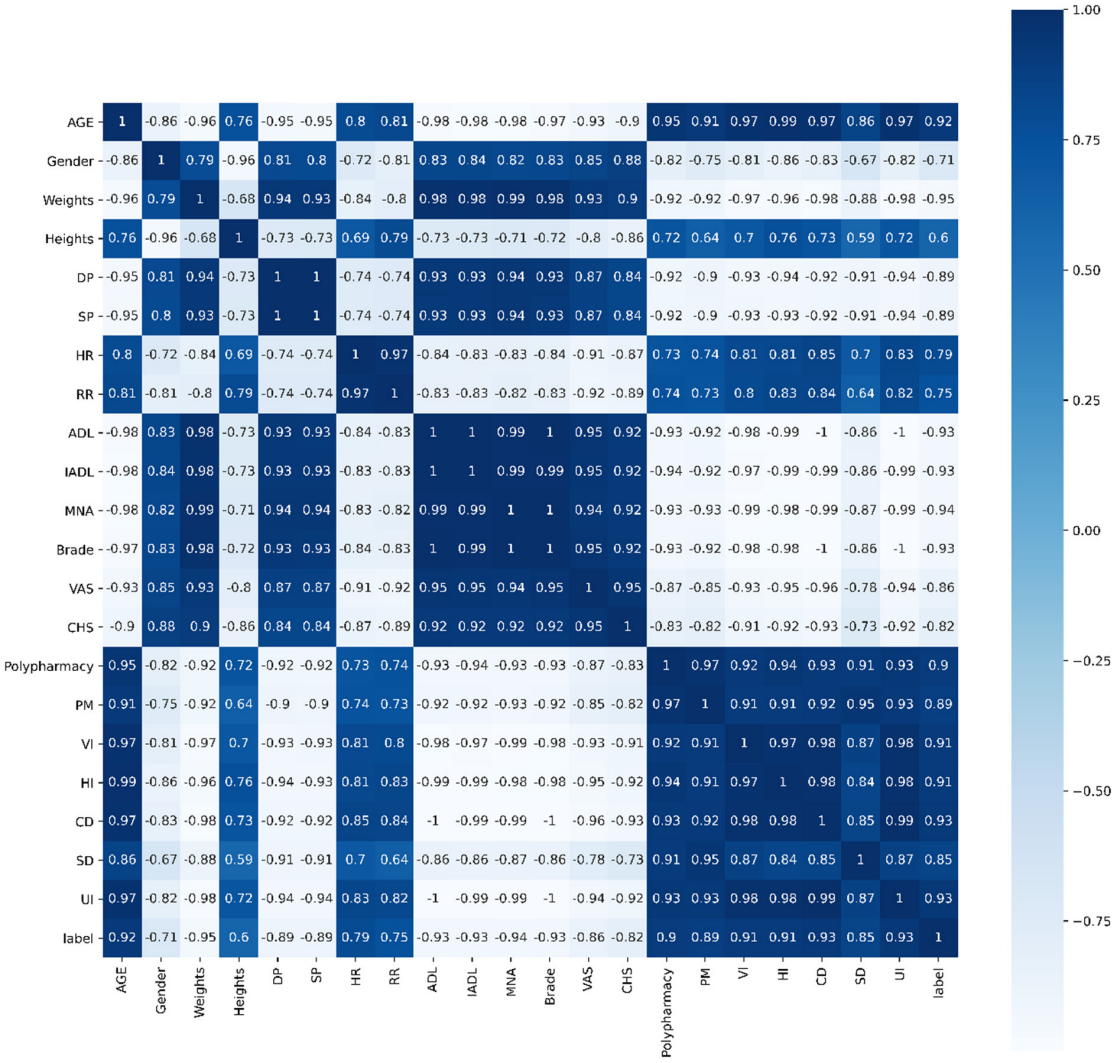
Spearman's correlation of all 21 features.

**Figure 10:**
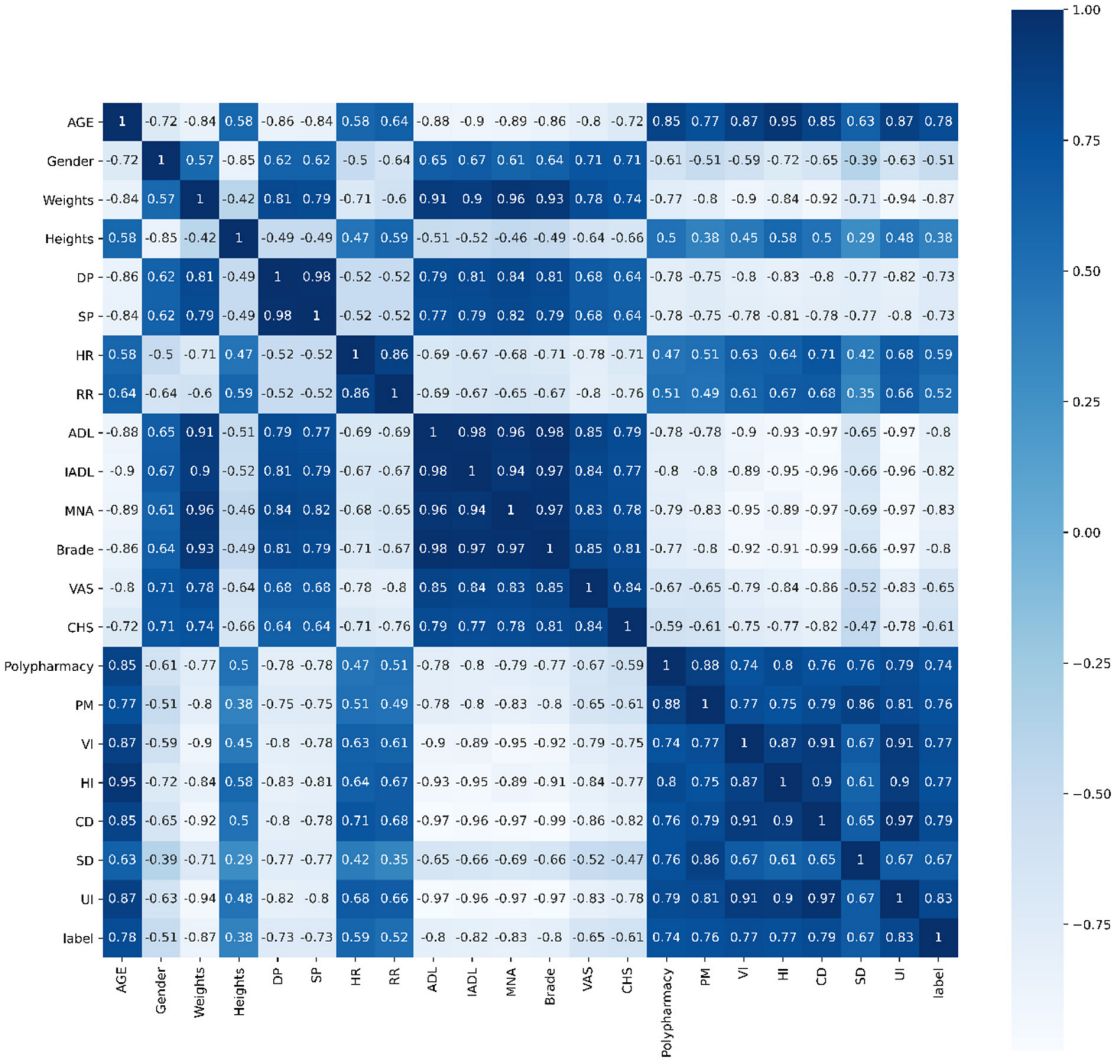
Kendall's correlation of all 21 features.

Pearson's correlation, Spearman's correlation and Kendall's correlation coefficient were used to analyze all features included in the study, as Figures 8–10. From Pearson's correlation, gender, weight, systolic pressure, diastolic pressure were highly related to ADL, IADL, MNA, Brade score, VAS and CHS from CGA. While age, heart rate (HR) and respiratory rate (RR) were highly related to polypharmacy, psychiatric medication (PM), visual impairment (VI), hearing impairment (HI), communication disturbance (CD), sleep disturbance (SD) and urinary incontinence (UI). Fall was positively related to age, HR, RR, polypharmacy, PM, VI, HI, CD, SD and UI, while negatively related to weight, diastolic pressure (DP), systolic pressure (SP), ADL, IADL, MNA, Brade score, VAS and CHS.

## Discussion

In this study we adopted data analysis combined with machine learning and deep learning to analyze general factors from EHR and CGA of hospitalized elderly patients. The model predicted the risk of falls, facilitating medical staff to make hierarchical management and fall prevention strategies to reduce the elderly both in hospitalization and subsequent falls. The aim of this work is to predict fall risks among hospitalized elderly patients using an approach of artificial intelligence. Results from our study revealed that the top 5 features of importance to predict fall are IADL, Brade score, ADL, age and systolic pressure. Findings suggest that healthcare professionals treating elderly should focus more on these 5 features, as they could present risks of future falls.

Results of our study are different from previous studies exploring fall risks among elderly based on analyzing electronic health records. Lindberg et al., reported that history of falls, age, Morse Fall Scale total score, mental status, unit type, gait/transferring and the number of high risk FRIDs are the most relevant factors across bagging, random forest, and boosting models (34). Ye et al. (40) used XGBoost algorithm to capture 157 impactful predictors into their final predictive model, and identified the top-5 strongest predictors of the future fall event as cognitive disorders, abnormalities of gait and balance, Parkinson's disease, fall history and osteoporosis. From the nurses' perspective, Jung et al., reported that dysuria and lower limb weakness are important risk factors predicting fall among elderly using the analysis of logistic regression model and COX PH regression model (33). Our study using CGA demonstrated that ADL and IADL are also two important risk factors of fall risk among the elderly.

Besides, we put different features to calculate accuracy in each model, and it revealed that with only 15 features, XGBoost model gave the highest accuracy. We believed that it's useful to use only 15 features to predict fall among elderly, because it's more difficult to collect data from elderly, especially data of function, frailty and emotional status. Future study is warranted to manage appropriate feature selection to predict fall in different population or different setting.

This is the first study ever done using CGA with machine learning to predict fall risk among elderly. CGA is a multi-dimensional, multi-disciplinary diagnostic and therapeutic process conducted to determine the medical, mental, and functional problems of older people with frailty so that a coordinated and integrated plan for treatment and follow-up can be developed (41). Nowadays, CGA is used widely and regarded as the gold standard for caring for frail older people in hospitals (42). CGA has also been used to identify risk of adverse events such as mortality, functional decline, surgical complications, and chemotherapy toxicity among cancer patients (43). CGA has been used in machine learning to better evaluate older patients with atrial fibrillation (44). Our results showed that CGA is a useful tool for fall prediction, especially for ADL, IADL and Brade score. Future study is warranted for identification and intervention for prevention of fall after machine learning prediction.

There have been multiple fall risk assessment tools such as Morse Fall Scale, STRATIFY and SPPB. However, these tools are with several disadvantages as follows: 1. Their specificity was relatively low, thus there could be some unidentified high risk elderly (45); 2. Not all hospitals use these tools as routine assessments, so the clinical usefulness is doubted; 3. These tools requires time for assessment and data entry, causing more documentation burden (46). In our model, measurements such as systolic pressure, diastolic pressure, height, weight, heart rate, etc. are easily measured. As for some functional assessments such as ADL and IADL that requires more clinician time, they are currently widely accepted assessments by medical facilities and much hospitals integrated them into routine care. Thus, we still believe our model can be applied to other hospital or healthcare organization to prevent future fall of elderly owing to its simplicity and accuracy. However, the assessment and documentation burden are still not solved. Under current development of machine learning and artificial intelligence, we believe that there will be a simple way to measure and predict functional disability of elderly, as some research already did (47).

Our study has some limitations. First, the investigation was limited to data from a single hospital, thus external validity should be interpreted with caution. Further testing our models on data from other hospitals in other regions is needed to establish external validity. Second, some objective data were lacking, such as albumin and hemoglobin levels, and these blood data are likely important factors for predicting fall. Future analyses should include such data for a better model. Third, only 21 features were analyzed to reach best model in the study, this may not reflect real condition of participants. However, we selected 21 features out of CGA and EHR after carefully discussion among the expert group, and we did analyze more features from CGA but the outcome was not promising enough as there were lots of data recording as 1 or 0 in CGA. Future study will be aimed to explore more appropriate features from CGA to reflect true condition of the elderly. Fourth, some important factors related to fall risk were not considered, such as caregiver-related factor. Future project should include those important factors to reach a better fall risk prediction. Fifth, our predictive performance is not as good as previous results. However, the prediction model was used by only 21 features. We believe our model can be applied to prevent future fall of elderly as an applicable and useful approach. We hope that our study could be a touchstone of future researchers interesting in this quality of life among elderly to put more emphasis on function limitations such as ADL and IADL limitation as they are also important feature fall risk from our results.

This is the first machine-learning based study using both electronic health records and comprehensive geriatric assessment to predict fall risks of elderly. Multiple risk factors of falls in hospitalized elderly patients can be put into a machine learning model to predict future falls for early planned actions. The prediction model was used by only 21 features. We believe our model can be applied to other hospital or healthcare organization to prevent future fall of elderly and improve their quality of life.

## Conclusion

We predicted fall risks among the hospitalized elderly by combination use of HER and CGA. We found that IADL, Brade score, ADL, age and systolic pressure are 5 important features in the prediction model. The accuracy rate of XGBoost evaluation reached 73.2% based on 21 features. Such a model can be a useful tool due to its simplicity and good accuracy.

In future adjustments of the model, there are several directions. First, we would like to screen the severity of chronic diseases, as chronic diseases cannot be quantified as only 1 or 0 in the model to represent true condition of elderly, to improve even more the accuracy of model prediction. Second, we will explore the application of feature selection in different machine learning models among elderly, because from our results, it was shown that feature selection was complicated as well as important. Third, we will perform validation in different settings, such as post-acute care department or long-term care facilities to validate our models.

## Data availability statement

The original contributions presented in the study are included in the article/Supplementary material, further inquiries can be directed to the corresponding authors.

## Ethics statement

The study was conducted according to the guidelines of the Declaration of Helsinki and approved by the Institutional Review Board (or Ethics Committee) of Taichung Veterans General Hospital (protocol code TCVGH-IRB CE20234A and date of approval: Aug 13, 2020). Written informed consent for participation was not required for this study in accordance with the national legislation and the institutional requirements.

## Author contributions

Y-TT and C-TY conceived of the study and supervised all aspects of its implementation. W-MC completed the analyses and drafted the content. EK and S-YL assisted with the study design and revised the content. Y-CW, W-CC, and Y-RL assisted with the statistical analysis and revised the content. All authors helped to conceptualize ideas, interpret findings, review drafts of the manuscript, contributed to the article, and approved the submitted version.

## Funding

This work was supported by Taichung Veterans General Hospital, Taiwan (Grant Number: TCVGH-T1097801 awarded to W-MC). The funders had no role in the design of the study, in the collection, analyses, or interpretation of data, in the writing of the manuscript, or in the decision to publish the results.

## Conflict of interest

The authors declare that the research was conducted in the absence of any commercial or financial relationships that could be construed as a potential conflict of interest. The handling editor W-JL declared a shared affiliation with the author W-MC at the time of review.

## Publisher's note

All claims expressed in this article are solely those of the authors and do not necessarily represent those of their affiliated organizations, or those of the publisher, the editors and the reviewers. Any product that may be evaluated in this article, or claim that may be made by its manufacturer, is not guaranteed or endorsed by the publisher.
