# Supplementary material for: A model for predicting fall risks of hospitalized elderly in Taiwan-A machine learning approach based on both electronic health records and comprehensive geriatric assessment
Source: Front Med (Lausanne). 2022 Aug 9;9:937216. doi: 10.3389/fmed.2022.937216 (PMC9398203; doi:10.3389/fmed.2022.937216)
Supplement: Supplementary file 1 [file Data_Sheet_1.docx]

Supplementary Material

## Supplementary Figures


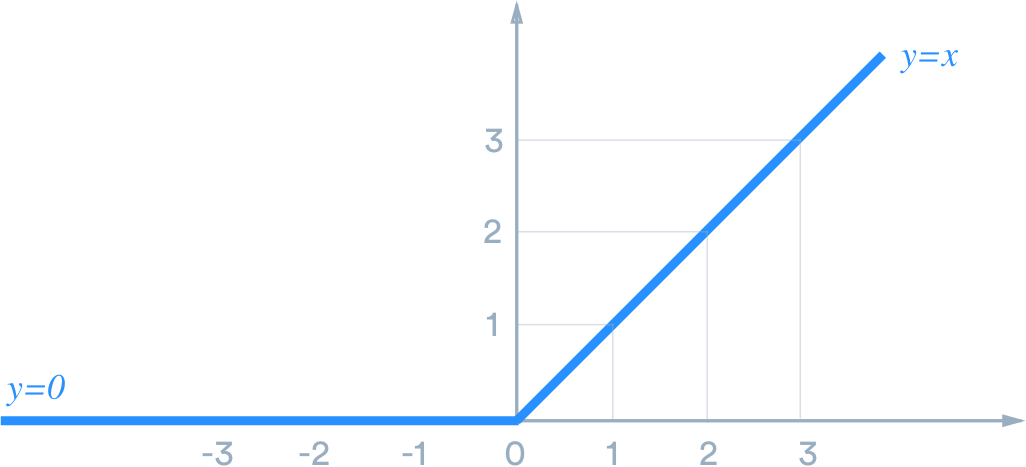


**Supplementary Figure 1.** ReLu function


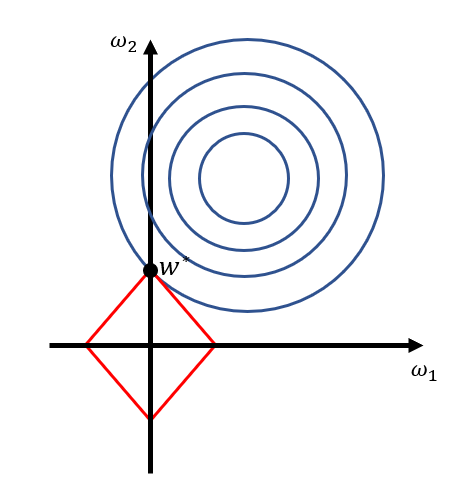


**Supplementary Figure 2.** L1 normalization


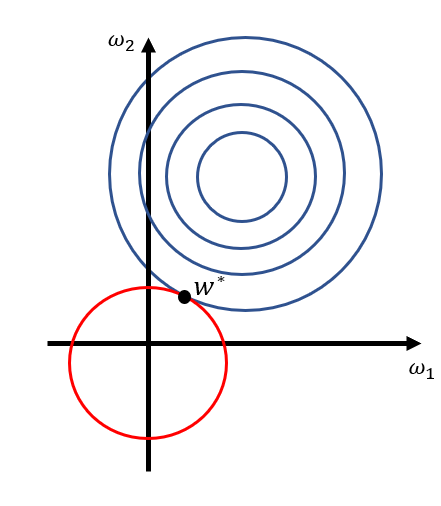


**Supplementary Figure 3.** L2 normalization


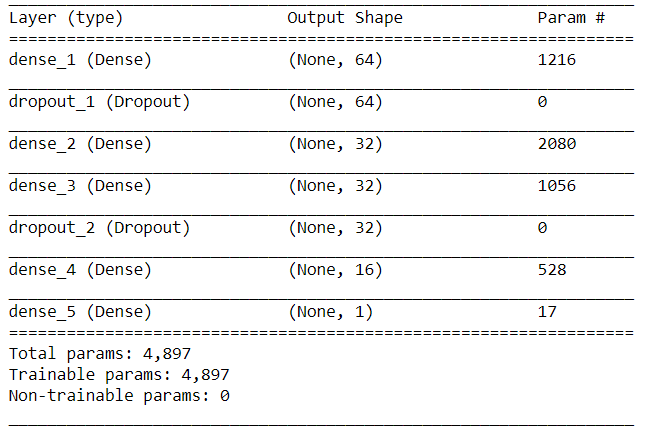


**Supplementary Figure 4.** Neural network layer

## Supplementary Tables

Supplementary Table 1. 95% CI for accuracy, sensitivity, specificity and auROC in XGBoost

| XGBoost | 95% CI |
| --- | --- |
| Val_accuracy | (59.5%-74.8%) |
| Test_accuracy | (58.2%-75.5%) |
| Train_accuracy | (71.8%-76.1%) |
| Val_sensitivity | (91.1%-100%) |
| Test_sensitivity | (91%-100%) |
| Train_sensitivity | (99.3%-100%) |
| Val_specificity | (0%-12.9%) |
| Test_specificity | (0%-11.8%) |
| Train_specificity | (9.8%-27%) |
| Val_auROC | (45.5%-53.6%) |
| Test_auROC | (47.7%-50%) |
| Train_auROC | (55.9%60.3%) |

Supplementary Table 2. Independent testing of XGBoost

| Independent testing | XGBoost |
| --- | --- |
| train Accuracy | 0.759091 |
| train Recall | 0.227941 |
| train F1 score | 0.369048 |
| train AUC | 0.812352 |
| Test Accuracy | 0.654545 |
| Test Recall | 0.027778 |
| Test F1 score | 0.05 |
| Test AUC | 0.503566 |
| Val Accuracy | 0.621622 |
| Val Recall | 0.024390 |
| Val F1 score | 0.045455 |
| Val AUC | 0.613066 |

Supplementary Table 3. Predictive performance based on the top 5 features in XGBoost

| Predictive performance | XGBoost |
| --- | --- |
| Accuracy | 0.707 |
| AUC_macro | 0.595 |
| AUC_micro | 0.726 |
| f1_score_macro | 0.518 |
| f1_score_micro | 0.707 |
| precision_score_macro | 0.688 |
| precision_score_micro | 0.707 |
| recall_score_macro | 0.55 |
| recall_score_micro | 0.707 |
